# Assessing the Equity of Accessibility to Urban Green Space: A Study of 254 Cities in China

**DOI:** 10.3390/ijerph19084855

**Published:** 2022-04-16

**Authors:** Yingxue Rao, Yi Zhong, Qingsong He, Jingyi Dai

**Affiliations:** 1 College of Public Administration, South Central University for Nationalities, Wuhan 430074, China; raoyingxue_ryx@163.com (Y.R.); 2020110098@mail.scuec.edu.cn (Y.Z.); 2 Research Center of Hubei Ethnic Minority Areas Economic and Social Development, South Central University for Nationalities, Wuhan 430074, China; 3 College of Public Administration, Huazhong University of Science & Technology, Wuhan 430074, China; 4 School of Environmental Science and Engineering, Huazhong University of Science & Technology, Wuhan 430074, China

**Keywords:** green space, accessibility analysis, justice, housing price

## Abstract

Urban green space has environmental benefits of purifying the air, reducing the heat island effect and providing the social and economic benefits of rest places and social platforms. An integrated and organized green space system is important for fully realizing the positive functions of an urban ecosystem. Previous studies have considered green space supply and demand, but few studies have examined large-scale, diverse and small-scale systems, making it difficult to conduct a comparative study of urban green space accessibility and equity under the same conditions (such as data sources and calculation methods). Using the two-step floating catchment area method, this study evaluates the equity of 254 urban green spaces in China within four ranges of accessibility: 1 km, 2.5 km, 5 km and 10 km. The study also considers urban house price in the research. The results show the following: (1) There are large differences in the accessibility of green space between different cities in China. Within the accessibility threshold of 10 km, the city with the most accessible urban green spaces has an accessibility level that is 27,813 times that of the city with the lowest accessibility. (2) Within the range of walking/cycling, there are significant inequalities in green space access in the 254 cities; the inequality of green space accessibility in most of the studied cities is at the “dangerous” level. (3) The two-step floating catchment area method indicates that the social superiority (high social class) represented by high housing prices is associated with a greater opportunity to access urban green space services. This paper highlights the main problems associated with the accessibility of urban green space in China and proposes targeted development recommendations. These recommendations provide a reference for urban managers to develop effective green space development policies and realize the optimal allocation of urban green space.

## 1. Introduction

Urban green space (UGS) refers to sites for planting trees, flowers and plants and supporting facilities. These spaces are essentially covered by green plants and endowed with certain functions and uses, including that of public green space, residential green space, traffic green space, ancillary green space, production protection green space and scenic area green space, located in a city or suburb. UGS purifies the air [1], protects biodiversity [2], reduces the urban heat island effect [3], and reasonably distributed and accessible UGS provides residents with sports venues, encourages residents to participate in physical exercise and ensures residents’ health [4,5]. In addition, UGS provide an attractive social and rest space, especially for large-scale vulnerable groups in the city [6], such as the elderly with poor mobility [7], children [8] and the homeless [9]. The optimal spatial layout of a functional network transportation system and UGS is a foundation for maintaining a balanced urban landscape environment [10]. Therefore, an integrated and organized UGS system helps realize the functional needs of an urban ecosystem.

Early evaluations of UGS focused on indicators such as UGS area and proportion and focused mostly on large parks. This approach, however, does not fully reflect the spatial distribution of the UGS system, lacks specific indicators to guide the spatial distribution of UGS and does not address the equity of UGS service functions. The continued advance of urbanization and urban population growth is intensifying the dynamic between UGS availability and residents’ well-being, which has also led researchers and urban planners to consider the rationality of green space layout and service fairness, especially in dense and compact urban areas [11,12,13]. The scope of ecological evaluations of UGS has gradually developed from a micro scale to a macro scale, with an increasing emphasis on ecological service functions and the spatial structure evaluation of green space [14].

Accessibility is used to quantitatively express the ability of residents to overcome obstacles such as distance, travel time and effort in order to reach a service facility or activity site [15]. The accessibility level of UGS has a profound impact on the frequency at which the urban poor, the elderly, children and the disabled use UGS and other public service facilities. Accessibility is an important indicator that reflects whether the natural services provided by UGS can be easily and fairly enjoyed by citizens. Introducing a series of indicators related to accessibility and equity into the functional evaluation of UGS can effectively represent and measure the effectiveness of UGS layout.

Most studies on the equity associated with UGS accessibility have been concentrated in developed countries such as Western Europe [16,17] and the United States [18,19]. However, urbanization in China is accelerating, with urban land area significantly expanding in recent decades [20]. In this context, it is particularly important to counterbalance land use, given the limited supply of public service facilities such as UGS and the increasing demand for these spaces by the people. However, several studies have been conducted in metropolitan areas, including, Hong Kong [21], Macao [22], Beijing [23], Shanghai [24] and Nanjing [25]. Together, these studies do not provide sufficient diversified urban samples and have not included systematic national research; as such, they do not address the heterogeneity of small and medium-sized cities and it is difficult to compare the accessibility and equity of UGS at different spatial scales. It is also difficult to discern the environmental equity issues behind the ineffective distribution of UGS. This highlights the need to study UGS accessibility and underlying equity at a large scale, in diversified settings, and at a small-scale in order to support the support the improved environmental justice associated with UGS in China.

## 2. Literature Review

### 2.1. Urban Green Space Accessibility and Equity

Spatial accessibility analysis is widely used to study medical services [26], educational resources [27], transportation [28], park green space [29,30] and other public services. For UGS, spatial accessibility refers to the convenience with which different social groups can arrive at the UGS from a certain place using a certain mode of transportation. Spatial equity refers to the analysis of the differences between different social groups in obtaining UGS, from the perspective of those who supply and demand the resources [31]. The main research methods adopted by scholars include the gravity model method [32], Gaussian-based two-step floating catchment area method [33,34], cost-weighted distance method [35] and network analysis method [36,37].

Several research results have reached consensus about the environmental and social benefits of UGS; however, many studies around the world show that there are great differences in the spatial distribution of green space in different areas of the city. These scholars have conducted a series of studies to explore the reasons behind these differences and to promote a fair distribution of social infrastructure.

In many cities in Europe and America, UGS tends to be spatially distributed in areas where there are more rich and white people, due to the increasing income gap and the expansion of immigration [33,38,39]. For example, Dai [33] evaluated the spatial accessibility of 890 green space samples in metropolitan Atlanta, Georgia; results suggest that the neighborhoods with a higher concentration of African Americans had significantly poorer access to green spaces (*p* < 0.05). Low income and non-white groups often dwell in places where public health challenges are most severe and there is often a relative lack of safe and well-maintained parks and other types of open space [40]. Moreover, these groups are unlikely to have effective representation in the decision-making process. They are also less likely to have the ability and resources to effectively apply for green areas [41]

In China’s complex and dense urban environment, there are significant differences in the distribution, structure and composition of UGS. Rapid urbanization takes up UGS, leading to reductions in size, increased space fragmentation and increased complexity of the space form; these cause social, economic and environmental problems [42]. For example, empirical studies found that from 1999 to 2013, 88.1 km^2^ of UGS in Dalian was converted to other land use and the UGS decreased by 29.4% [43]. Meanwhile, the effective utilization rate of UGS in China needs to be improved. A study in Chengdu found that the UGS coverage rate in in 2018 reached 37.71%, but the effective utilization rate was below 27.49% [44]. If a park is well built but has poor accessibility, it cannot provide enough entertainment and ecosystem services functions for residents. The landscape fragmentation is relatively high and the range of human activities is not rational, leading to poor balance among multiple ecosystems [44]. In addition, urban land transfers mainly occur in the periphery of cities and urban fringe areas have experienced significant environmental and social changes. In China, research by Shenyang [35] and Shanghai [24] have observed the problem of poor UGS accessibility in urban fringe areas.

Equity in the patterns of UGS may be influenced by the functions of administrative districts, the distribution of residential land, resident travel distance and urban development policies. Many studies have found a mismatch between UGS and the population in China, with an unbalanced and unfair distribution of green space resources for resident enjoyment [23,34,45,46]. It is also highlighted that the equity problem of UGS is essentially caused by the scarcity of UGS resources [45,46] and inadequate provision may be caused by factors, such as dry cold climate and urbanization patterns (e.g., infill development) [46]. In addition, the green gentrification of Chinese cities may exacerbate this inequity [47].

To alleviate the irrationality of UGS distribution, some scholars have proposed that planners promote green space intervention in small scale and scattered locations, rather than pursuing large UGS projects that geographically concentrate resources and initiate gentrification [41]. A study in Macau concluded that constructing new micro UGS in high-density cities would increase the coverage of UGS at a lower cost. Although some small UGS have limited recreational uses, they provide areas of ecological and environmental benefit where there is a high degree of urbanization [22]. He et al. [47] examined the fairness of the supply of the different green spaces in Wuhan for the entire population and different social groups and also recommended improving UGS for residents through micro-scale green spaces, such as pocket parks, on the city’s outskirts. Some studies also note that measures such as strengthening traffic construction to reduce the communication cost between residential land and UGS can also improve somewhat the fairness of urban green space access [37,45].

### 2.2. Limitations of Existing Research and Innovations of This Paper

A review of previous studies indicates that the accessibility index of UGS can reflect the distribution of UGS facilities when integrated into a UGS evaluation system. This leads to the following hypothesis: in the context of rapid urbanization, imbalanced and unfair UGS accessibility has become increasingly prominent. However, previous studies have some limitations and room for improvement:

(1) Previous research has mainly focused on European and American cities. There have been few studies in the context of developing countries; and for China, there have been few large-scale diversified urban sample analyses.
(2) The selection of different research scales across previous studies may lead to different results that cannot be compared. Generally, a smaller research scale is associated with higher data accuracy. Therefore, starting from the community scale, this study provides a comprehensive look at the overall green space pattern of the research region. This allows for the identification of the fine units lacking a supply of green space and leads to research conclusions that are more accurate and have practical value.

## 3. Materials and Methods

**Figure 1:**
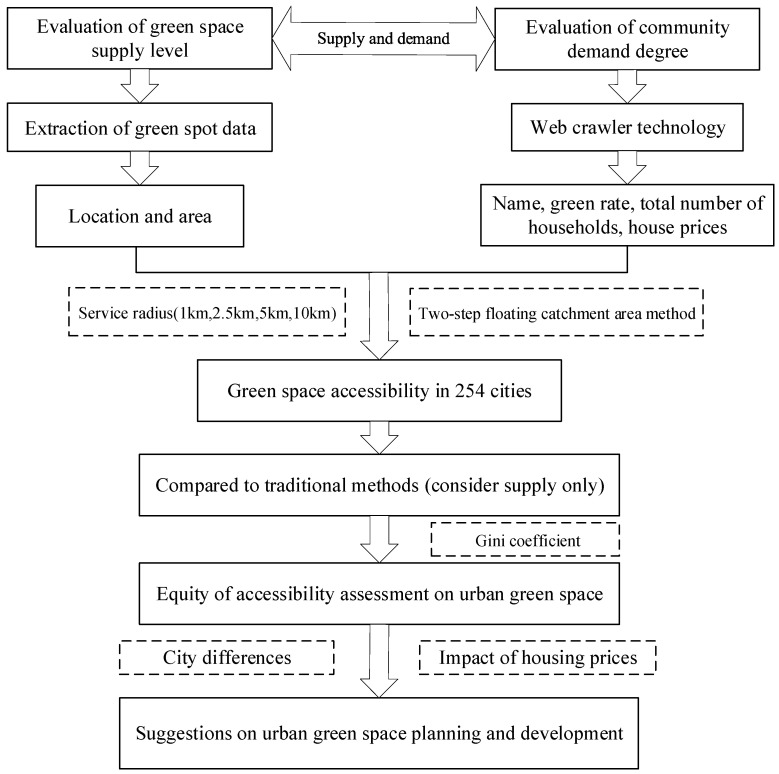
The research framework for this study.

This paper proposes the following research questions: What is the accessibility, and equity of accessibility, for Urban green space (UGS) in 254 cities in China? Based on the background above, Figure 1 presents the research framework. To fill current research gaps, this study applies the two-step floating catchment area method (2SFCA), combined with the Gini coefficient, and uses community scale UGS data from 254 cities in China to build a UGS accessibility fairness evaluation system. The goal is to comprehensively and accurately explore the differences in spatial fairness with respect to the accessibility of different UGS resources.

### 3.1. Methods

The 2SFCA method focuses on supply and demand areas, combined with a search radius. It assumes that residents in a search area have equal access to resources, but also considers the characteristics of service capacity attenuation with distance. Evaluating spatial accessibility can reflect the differences in public service facilities in different regions, with reliable results [48]. This method considers the interaction between green space and population, allows people to estimate the number of green spaces that residents can enter by walking, cycling, or driving and helps to identify shortage areas in order to clarify gaps.

The method can be divided into three steps.

The first step is to calculate the ratio of UGS to residents (*R_j_*) to determine the degree of busyness with respect to the supply point, as Equation (1):

(1) $$R_{j}=\frac{S_{j}}{\sum_{k\in \left\{d_{ij}\leq d_{0}\right\}}G\left(d_{ij},d_{0}\right)H_{i}}$$

where *i* is the demand point (i.e., residential location); *j* is the supply point (i.e., green space location); *S_j_* is the service capacity of green spot *j* (i.e., the total area of green space in the search area, m^2^); *d_ij_* is the distance between demand point *i* and supply point *j*, expressed by distance (m); *d*_0_ is the search radius, which is the effective service radius of the facility; and *H_i_* is the number of households at location *i* whose centroid fall in the catchment (i.e., *d_ij_* ≤ *d*_0_) from green space location *j*. The expression $G\left(d_{ji},d_{0}\right)$ represents the distance attenuation function within the search radius *d*_0_; and *G*(*d_ij_*) can be divided into the form of piecewise attenuation by distance, the form of distance attenuation function of gravity model (such as power function or exponential function), the form of kernel density, or the Gaussian form [49]. In this paper, the Gaussian function is used as *G*(*d_ij_*) within the search radius of 2SFCA and the formula is expressed as:

(2) $$G\left(d_{ji},d_{0}\right)=\{\begin{array}{cc} \frac{e^{-\left(\frac{1}{2}\right)\times \left(\frac{d_{ji}}{d_{0}}\right)}-e^{-\left(\frac{1}{2}\right)}}{1-e^{-\left(\frac{1}{2}\right)}} & if\leq d_{0}\\ 0 & if\;d_{ji}>d_{0} \end{array}$$

The second step is to calculate the accessibility Ai of UGS.

(3) $$A_{i}=\sum_{l\in \left\{d_{il}\leq d_{0}\right\}}G(d_{il},d_{0})R_{l}$$

*A_i_* represents the accessibility of demand point *i* calculated using 2SFCA. It represents the average facility resources accessible to each demander at demand point *i*; and l represents all green space within the catchment area of population location *i*.

The third step is to use the Gini coefficient to measure the fairness of urban green space layout, defined by evenness of access. The Gini coefficient is an indicator used to reflect the degree of equality of distribution and is calculated according to the Lorenz curve. It is a statistical indicator that quantitatively and accurately reflects the degree of fairness in social income distribution. The coefficient ranges from (0–1); a higher value is associated with a higher degree of inequality of access. Table 1 presents the criteria for determining the level of inequality.

(4) $$GC=\sum p_{i}y_{i}-2\sum {\left(\sum P_{i}\right)}^{’}y_{i}+1$$

where *GC* is the Gini coefficient; *p_i_* is the proportion of the number of households; in group *i* to the total number of households; ${\left(\sum P_{i}\right)}^{’}$ is the proportion of the cumulative number of households from group 1 to group i in the total number of households; and *y_i_* is the ratio of accessibility value to total value.

In addition, in 2SFCA, it is very important to establish the optimal search radius, labeled as *d*_0_. A selection range that is too small will not support the reasonable construction of UGS; a selection range that is too large may lead to the poor utilization of park green space. Considering the imbalance of urban development in China and the travel modes of walking, cycling and driving, referring to the selection of accessibility thresholds of Wu et al. [23], this study tested four thresholds, ranging from 1 km to 10 km and established a research focus on walking/cycling (1 km and 2.5 km) and driving (5 km and 10 km).

### 3.2. Study Area and Data Sources

This study is based on cities of prefecture-level and above to evaluate the use and distribution of UGS by analyzing the accessibility and equity of access to green space in China. Considering the availability of relevant data, 254 cities were selected as the study area. The research area for each city was defined based on constructed land data, extracted from the national land use cover database (https://www.resdc.cn (accessed on 11 December 2015)) for 2015, at a spatial resolution of 30; this was done to determine the validity of the evaluation results.

The UGS data were collected from green space map spot data [50]; parks were defined as including scenic spots, urban parks and green spaces. The UGS selected in this study all exceeded 1 hectare in size, covering a total area of 129,012.84 hectares.

Community data were collected from China’s largest real estate rental and sale service network platform, Anjuke (www.anjuke.com (accessed on 9 October 2017)). Web crawler technology was used to capture the community information listed on the website in July 2017. Data points include community name, community coordinates (latitude and longitude), total number of households and housing price. According to the principle of completeness of data attributes, a final count of 80,341 plots were obtained after removing plots with missing attributes. The final spatial distribution of plots was obtained after spatializing the plots using the coordinate information. Table 2 shows the basic data on the green space and residential areas of the 254 cities.

## 4. Results and Analysis

### 4.1. Accessibility of UGS

**Figure 2:**
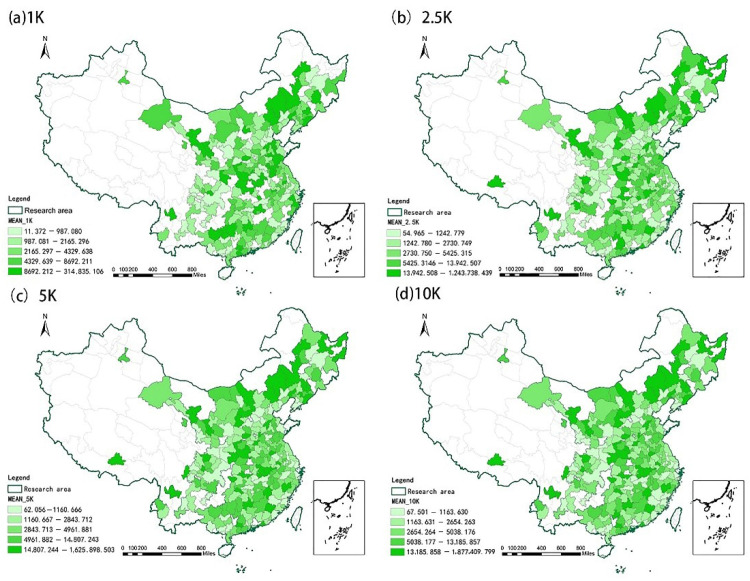
Changes in the spatial accessibility in the study area when (**a**) *d*_0_ = 1 km; (**b**) *d*_0_ = 2.5 km; (**c**) *d*_0_ = 5 km; and (**d**) *d*_0_ = 10 km.

Before measuring the equity of UGS accessibility, we first evaluate UGC accessibility. The average UGS accessibility of 254 cities within the radii of 1 km, 2.5 km, 5 km and 10 km is shown in Figure 2. UGS accessibility is generally poor in some cities in China, such as Suihua City and Yan’an City, within the four search radii thresholds. We hypothesize that this is related to the relatively scarce green land resources in China. The average amount of green space in Chinese cities is in short supply relative to the current standards, such as that of at least 8 m^2^ for National Garden City evaluation; the green space coverage rate in most cities is low; and the green space level per capita is poor [51]. Meanwhile, this study shows that the accessibility level of UGS in 254 cities is highly variable, highlighting the need for in-depth investigation and discussion from the perspective of fairness.

This study found that, within a search radius of 1 km, the top 10 cities in the average community UGS accessibility level have accessibility indices as follows: Jinchang 314,835.11, Dandong 154,600.69, Chengde 72,205.61, Guyuan 52,649.65, Huangshi 49,131.62, Wuwei 49,055.62, Fushun 44,696.62, Baicheng 32,957.54, Binzhou 32,235.85, and Bengbu 25,974.76. Within the search radius of 2.5 km, the top 10 cities with respect to the average community UGS accessibility level are: Jinchang 1243,738.44, Jiamusi 263,712.63, Huangshi 112,586.96, Datong 108,948.04, Ulanqab 92,910.61, Jingzhou 81,811.59, Baicheng 75,654.74, Yunfu 61,697.61, Tieling 60,323.73, and Chengde 58,157.78. Within a search radius of 1 km and 2.5 km, the accessibility of some cities is 0, such as Lishui, Chuzhou, Ningde, Yingtan, Fuzhou, Shiyan and Ya ‘an. Within a search radius of 5 km, the highest accessibility index value in Jinchang is 1625,898.50 and the lowest value is for Suihua, at 62.06, representing a difference of 26,199 times. Within the search radius of 10 km, the highest accessibility index value in Jiamusi is 1877,409.80 and the lowest value in Suihua is 67.50, a difference of 27,813 times. These types of regional differences have also been found in Europe [52]. Finally, this study found that when the search radius increased, the accessibility level of UGS improved overall. The improvement in travel mode helps encourage UGC accessibility, but is more inconvenient for vulnerable urban groups.

**Figure 3:**
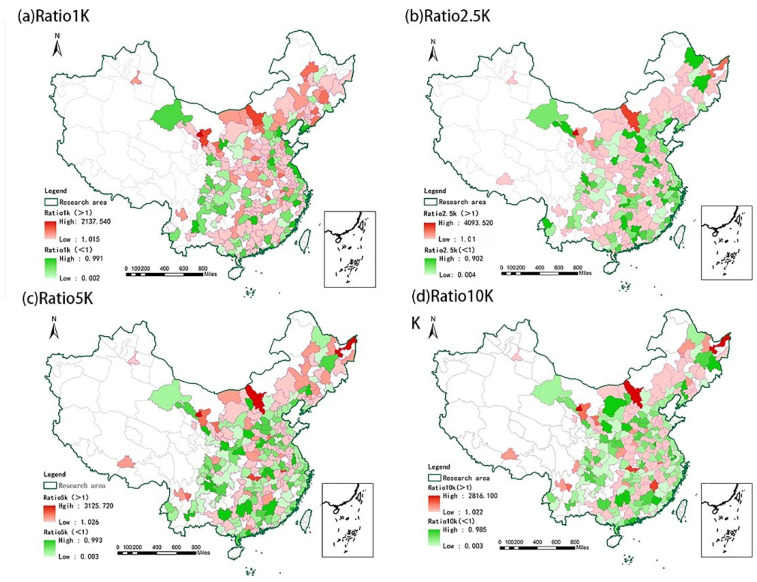
Comparison of 2SFCA and traditional accessibility method when (**a**) *d*_0_ = 1 km; (**b**) *d*_0_ = 2.5 km; (**c**) *d*_0_ = 5 km; and (**d**) *d*_0_ = 10 km.

To further explain our research method, we compared the 2SFCA, which considers supply and demand balance, with the traditional accessibility method, which considers supply, but not demand. This is measured using the ratio of the average accessibility calculated using 2SFCA to the average accessibility calculated by traditional methods. When the ratio exceeds 1, the traditional method underestimates the real accessibility of UGS, otherwise it overestimates it. Figure 3 shows the distribution of the ratio of the two accessibility measurement methods under the four search radii. We found that, with an increase in the search radius, the proportion of cities with ratio exceeding 1 changed from 58.66%, 53.54%, 40.94%, to 39.76% for the radii of 1 km, 2.5 km, 5 km, 10 km, respectively. Therefore, based on the data from 254 cities, when the search radius is small (1 km and 2.5 km), the traditional method tends to underestimate the real accessible value associated with UGS. In contrast, when the search radius is expanded to 5 km and 10 km, the traditional method tends to overestimate the real accessible value associated with UGS.

To better describe the differences between the two methods, we classify those cities with a ratio greater than 1 into one group and those with a ratio less than 1 into one group. We use a *t*-test to discuss whether there are significant differences in green area and average house price between the two groups. Table 3 shows the significant results, which indicate that there are significant differences in green area and the average house price between the groups. In general, cities with ratio >1 have a higher green area and higher average house price. In this case, the traditional method of only considering supply without considering demand underestimates the real accessibility of UGS, which could lead to a wasted supply of UGS (more than is needed to meet accessibility needs).

### 4.2. Accessibility Equity of UGS

**Figure 4:**
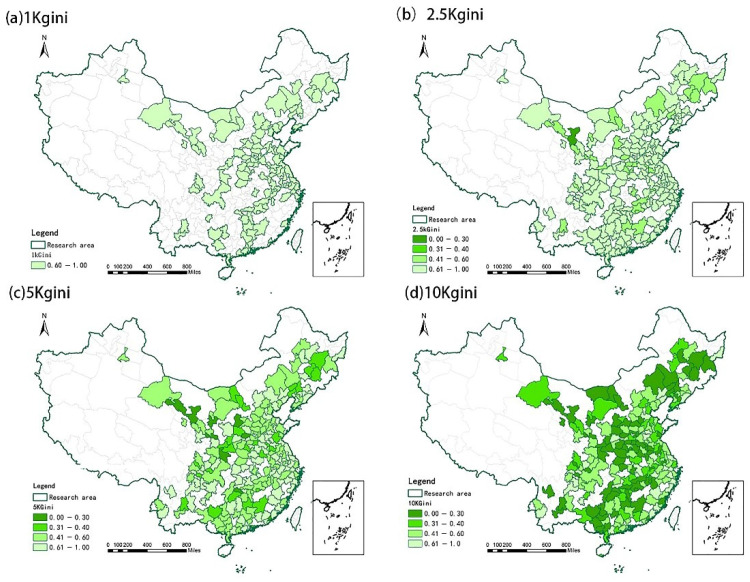
The Gini coefficient of spatial accessibility of the study area when (**a**) *d*_0_ = 1 km; (**b**) *d*_0_ = 2.5 km; (**c**) *d*_0_ = 5 km; and (**d**) *d*_0_ = 10 km.

We calculated the accessibility level at four distance thresholds (1 km, 2.5 km, 5 km and 10 km) for each community and then calculated the Gini coefficient of UGS accessibility according to the accessibility of multiple communities within each city. Due to the small number of community samples from a few participating cities, the accessibility of those cities is zero. To objectively consider the research results, we excluded these areas. Figure 4 shows a Gini coefficient distribution of UGS accessibility in China when considering the search radii of 1 km, 2.5 km, 5 km and 10 km. In general, UGS accessibility in China is highly unfair with respect to access. Within the search range of 1 km and 2.5 km (walking/cycling), the Gini coefficient of UGS accessibility is mostly within (0.6–1), that is, the inequality is at the “dangerous” level; the distribution gap of green space resources is wide; and the supply-demand relationship is out of balance. Within the search range of 1 km, the top 10 cities with respect to the Gini coefficient for UGS accessibility are: Xuzhou 0.7998; Foshan 0.7997; Qingyuan 0.7992; Zibo 0.7991; Heze 0.7991; Zhangjiakou 0.7991; Chizhou 0.7989; Xinxiang 0.7988; Wuhu 0.7988; and Zhuzhou City 0.7988. Within the 2.5 km search range, the top 10 cities with respect to the Gini coefficient for UGS accessibility are: Shaoxing City 0.7998; Yibin 0.7996; Maoming 0.7996; Langfang 0.7992; Zhaoqing 0.7992; Shangqiu 0.7990; Qinzhou 0.7989; Xuchang 0.7984; Chengde 0.7983; and Quanzhou 0.7981.

When the search radius is changed to 5 km and 10 km (driving), the equality of UGS accessibility gradually improves. Within the search range of 5 km, the Gini coefficient is mainly (0.4–0.6) and the inequality is mainly at the “warning” level. Within the search range of 10 km, the Gini coefficient is mainly (0–0.3), but there are still 33 cities in the “dangerous” level of UGS accessibility equality. The top 10 cities with respect to the Gini coefficient for UGS accessibility are: Ezhou 0.7779; Huanggang 0.7772; Meishan 0.7615; Anshun 0.7478; Lincang 0.7376; Langfang 0.7310; Jixi 0.7292; Huangshi 0.7269; Taizhou 0.7236; and Ziyang 0.7224.

This study’s results found that, within the radius associated with walking to green space, the inequality of green space accessibility in most cities in China is serious. This may create challenges for the elderly and children, who find this to be a less convenient way to travel. In addition, when the search radius is 10 km, there are still some cities with “dangerous” inequality of UGS accessibility. This also reflects the serious scarcity of green space resources in some cities in China. Overall, the results of our empirical analysis show that the distribution of UGS in China is not reasonable from the perspective of resident accessibility.

### 4.3. The Relationship between UGS Accessibility Equity and Housing Price

**Figure 5:**
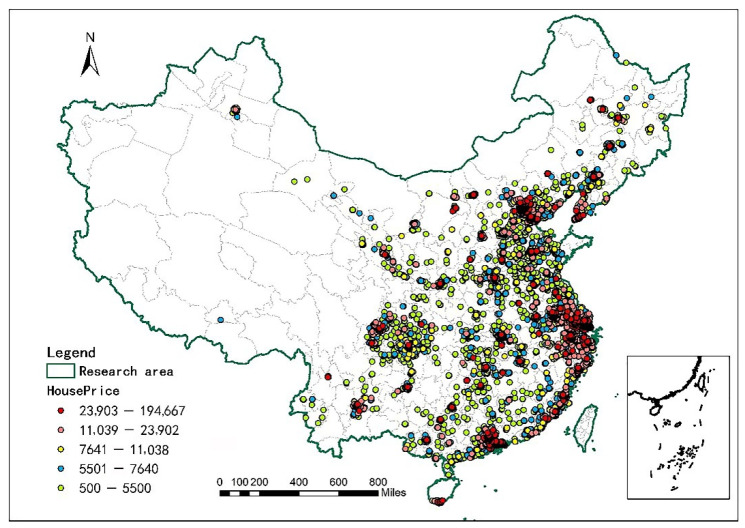
Location and average house price of the study area.

To further analyze the equity problem of UGS distribution, we apply an ordinary least squares (OLS) model to discuss the relationship between urban housing price and equity in UGS accessibility. Specifically, we divide the average housing price of cities into five levels and discuss the relationship between the housing price and the Gini coefficient of UGS accessibility at different threshold levels. Table 4 shows the results of the regression model. Figure 5 shows the average housing price of the 254 cities included in the research system.

The results of our empirical analysis show that, based on the sample data of participating urban communities, the relationship between average housing price and Gini coefficient of UGS accessibility is more significant in urban agglomerations where the average housing price level is between 11,039–194,667 yuan/m^2^. The cities with average house prices of 500–11,038 yuan/m^2^ are mainly located in the western and northeast regions of China, where there are poor economic conditions. Due to the relatively single urban form of these cities, which are inclined toward the “monocentric“ form and limited in green space and the number of community samples, the significance of the results is not strong.

For cities with an average house price between 11,039 and 23,902 yuan/m^2^, within the four thresholds of 1 km, 2.5 km, 5 km and 10 km, there is a significant positive relationship between house price and the Gini coefficient of UGS accessibility. In other words, from the perspective of providing rest services for residents, the higher the average house price is, the more unfair the UGS accessibility with respect to access. These cities are mainly located in the southeast coastal areas, which have better economic conditions in China. Residents are more willing to pay additional money for the comfort of their living environment and there is a more significant effect from the appreciation of the invisible value of urban parks on nearby houses. The higher the income level of cities, the more significant the difference in the accessibility of urban residential green space due to different income levels. At the same time, in these cities, the rise of residential prices also encourages park development, driving the surrounding traffic and real estate development.

At higher house prices (23,903–194,667 yuan/m^2^), the significance changes. In these cities, under the 1 km threshold, a higher average house price is associated with less fairness with respect to UGC accessibility. However, as the search radius expands, within the 2.5 km, 5 km and 10 km thresholds, a higher house price is associated with increased fairness in UGC accessibility. These cities are generally located in China’s most developed, super first-tier cities; most of the new residential areas have good green space and supporting fitness facilities. This somewhat offsets the impact on the surrounding housing prices, where urban parks are less attractive for residents. However, under the 1 km threshold, as the housing prices rise, UGC accessibility in these cities becomes more unfair. In the overall range, the better the economic development of the city is, the more reasonable the infrastructure layout is. The investment in the construction of green space is also higher and the planning of parks is relatively reasonable.

## 5. Discussion

### 5.1. Comparison to Previous Studies

The spatial equity of UGS is an important way to assess the balance of supply and demand between green space and citizens. It is also an important part of UGS research and planning decision-making. Optimizing the UGS system, improving the urban living environment and realizing social equity have become topics of significant focus for many scholars [53,54].

From a methodological perspective, to assess the environmental justice dimension of UGS accessibility in 254 cities in China, this method has important advantages. (1) It combines supply with demand, focusing both on the distribution of green space from the supply side and the accessibility of green space resources by residents on the demand side. This captures the differences in access to green space facilities in different regions. (2) This method captures the degree of reasonableness with respect to UGS distribution. Therefore, the framework can effectively reveal the accessibility of UGS and the rationality of the allocations, to better explore green space distribution justice problems in the process of urban development. This method can be applied to the practice of urban public infrastructure distribution, providing a theoretical basis for adjusting UGS layouts and providing a decision support tool. In addition, the results also reflect somewhat the possible drawbacks of traditional methods; that is, in cities with a relatively high total area of green space and average housing price, it is easy to underestimate the real accessibility of UGS, possibly leading to wasted supplies. In contrast, in the cities with a relatively low total area of green space and low average housing prices, it is easy to overestimate the real accessibility of UGS, which may lead to a lack of supply.

The location of UGS is affected by many factors, such as mode of land use, regional physical and geographical conditions, urban historical evolution, park funding mechanism and green space planning concepts [55]. At the same time, under the market mechanism, UGS also reflects certain positive externalities. Since the approach used for physical housing distribution in Chinese cities changed in 1998, China’s housing system has also changed from a system of welfare distribution to one of monetary distribution. When the accessibility or availability of facilities is strongly connected to a location or property rights associated with housing, urban public facilities can significantly impact the capitalization of housing [56]. As a result, more affluent people enjoy convenient public facilities and well maintained “green assets,” while low-income groups are more likely to live in municipal subsidized houses and social resettlement houses with smaller living space, a lower greening degree and older facilities [57]. This results in green gentrification [58]. The uneven distribution of UGS resources caused by income conditions has been found in Beijing [23], Harbin [59], Nanjing [60], Shenzhen [61] and Guangzhou, China [62]; Melbourne, Australia [63]; Warsaw, Poland [64]; and Seoul, South Korea [65]. Specifically, the value-added effect of green landscape on the surrounding housing prices leads to the disadvantage for low-income families in obtaining green land resources, resulting in a mismatch between population and green land and environmental justice problems.

Consistent with other studies [23,59,60,61,62,63,64,65], our study found that there is spatial inequality between supply and demand of UGS; and the social superiority represented by a high housing price is more likely to be associated with having access to UGS services. Moreover, with the support of a large scale of coverage and a large sample size, this study also reveals differences in the accessibility and fairness of UGS between regions and quantifies the relationship between regional housing price and the degree of equality with respect to regional UGS accessibility. This provides a further understanding and empirical basis for the distribution and layout of UGS.

In contrast to many European and American countries, China has different national conditions and a different development background. (1) The racial issue is not the focus of China’s environmental justice research. The problem of urban environmental justice in China is related more to the uneven distribution of green space and regional differences of population growth. (2) UGS planning and construction in China is led by the government. Based on national conditions and development needs in different periods, the requirements for UGS planning are also different. China is currently in a period of constructing an ecological civilization, with more complex requirements for supporting the ecological functions provided by green space [66]. The connection between urban and rural green space needs to be strengthened. As such, the Chinese government’s initiatives in the areas of mandates and the responsibilities of the Ministry of Land and Resources, for territorial spatial planning and new or updated laws, guidelines and standards, deserve special attention [66]. (3) As a highly populous country, China has a high demand for UGS and space utilization of UGS is more extensive and more frequent. (4) Advancing UGS construction is a comprehensive undertaking and China has made many attempts [67]. However, developed countries in Europe and America started constructing UGS systems earlier and invested more in theory and practice. This theoretical exploration and practical attempts at UGS system construction had costs and achieved success [68]. China should learn from the advanced experience of developed countries and develop a path that aligns with the country’s unique characteristics.

In the past, the rapid development of the social economy, regardless of the ecological environment, led to the lesson that destroying the ecological environment, and the excessive exploitation of natural resources, are related to ongoing ecological, natural and environmental crises. The rapid development of the city has led to the occupation of significant land resources, including a large number of green space resources [42]. In this respect, we recommend that cities seeking economic growth focus on rapid urban development and continuous economic transformation. A balanced approach will help achieve a winwin situation for both the economy and the urban environment in a time of steady economic growth. For the cities whose economy has developed to a certain level and whose urban construction has basically taken shape, it is important to focus at a deeper level, starting from the reasonable planning of UGS, social equity and other aspects. This will help establish a green city with low-carbon cycles, a suitable mix of people and land and sustainable development.

It is worth noting that the concept of a “compact city” has emerged as a planning method for sustainable development in areas with increasing urban population. Through managed densities and compact buildings, the compact city tries to offset the negative effects of urban expansion, such as ineffective land use and related environmental problems [69]. However, urban densification, including consolidation and filling development, may threaten UGS. This is because of the loss of public and private green space in cities due to densification measures, the risk of insufficient green space supply in densified areas and the risk of low green space planning priority in the context of development [70]. Similarly, an evaluation of the impact of urban form on UGS structure in 262 cities in China found that UGS landscape is often highly segmented by roads, leading to spatial fragmentation of UGS [71]. In this regard, we recommend that, during urban development, planners should consider the coordination of different elements, especially the protection of urban green patches.

**Figure A1:**
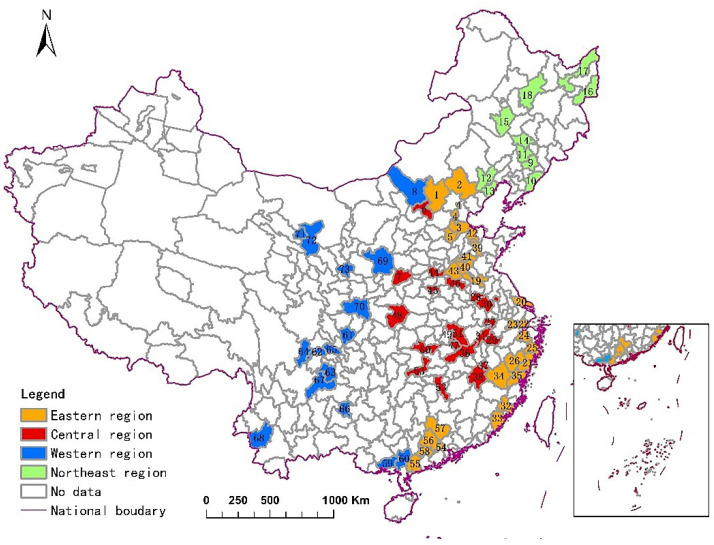
The city locations discussed in this paper (see the city names in the Appendix A).

To more intuitively represent the spatial distribution of green space resources in 254 cities, we recommend first focusing on the cities with Gini coefficients in the “dangerous” level (>0.6), within the 10 km search range. In 254 cities, this represents 12.99% of cities, including Ezhou, Huanggang, Meishan, Anshun, Lincang, Langfang, Jixi, Huangshi, Taizhou, Ziyang, Maoming, Chuzhou, Yuncheng, Quanzhou, Wenzhou, Shaoxing, Nanchong, Huludao, Tai’an, Huangshan, Yibin, Jining, Cangzhou, Huzhou, Zhaotong, Zhangzhou, Nantong, Foshan, Chaoyang, Jiaxing, Yiyang, Dandong and Nanping. In addition, within the search radius of 10 km, Suihua, Yan’an, Qinzhou, Yulin, Siping, Ningde, Hengshui, Hanzhong, Chaoyang, Jiujiang and other cities with poor accessibility. Table A1 and Figure A1 show the specific locations of the cities mentioned in this paper. In this regard, we recommend that extra attention be paid to the future construction and development of UGS in these cities. At the same time, we should also focus on cities with Gini coefficients in the “warning” level within each search radius.

### 5.2. Implications for Urban Green Space Planning and Management

UGS provides important public infrastructure in a city. To effectively allocate this, multiple subjects in the city need to engage in analyzing the reasons why the supply and distribution of UGS do not match the distribution of different groups and then develop corresponding measures based on that analysis. This study’s results found that, overall, the main problems of UGS in China are as follows. First, the accessibility level of UGS is poor and there are great differences between cities. Second, the distribution of UGS resources is unreasonable and different income groups have different levels of access to UGS. This highlights the need to enhance the concept of environmental justice and protect vulnerable groups who enjoy UGS resources. Third, regional development tends to increase somewhat the inequity of UGS accessibility, especially in urban agglomerations with an average house price of 11,039–23,902 yuan/m^2^.

Given these overall findings, we make five development recommendations.

First, local government should increase investments in UGS, improve the efficiency of UGS supply and develop strict regulations to provide basic investment and institutional guarantees for the construction of a regional ecological civilization. This would strengthen the city’s competitiveness and attractiveness. At the national level, poor cities with UGS deficits should be supported with construction funds. At the local level, especially in the cities of Western and Northeast China, local governments should both increase economic construction and address the problems of environmental justice faced by cities. At the government level, appropriate interventions are needed to balance the distribution of urban infrastructure investment and prioritize urban vulnerable groups when constructing UGS, in order to respond to the need for inclusive cities.

Second, we encourage the construction of small and scattered UGS within cities. First, this can help ensure the fair distribution of UGS and improve the inequality of access within 1 km despite scarce urban land resources. Second, this construction can expand the distribution of UGS, avoid UGS becoming the focus of real estate developers and alleviate green gentrification. Moreover, the construction cost of small green space is low and economical, both protecting residents’ rights and interests in green space resources and reducing the investment of construction funds. Finally, these measures would help purify urban air, increase air humidity, decrease the heat island effect and maintain the ecological environment.

Third, it is important to maximize opportunities created by the renovation of shrinking cities to convert low efficiency industrial land and brownfields into green spaces after demolition. This can improve the level of UGS, avoid wasting urban land and achieve urban renewal. Further, these inefficient industrial lands are mostly in areas with poor urban development; therefore, converting them to green spaces can effectively mitigate the problem of urban green gentrification, protect green spaces in areas with poor economic development and alleviate the unfair distribution of UGS.

Fourth, we encourage improvements in the mechanisms used to build new equitable systems of urban public resources, to enhance the opportunities for public participation in the construction of urban infrastructure and to ensure the public’s right to speak.

Finally, it is important to strengthen the synchronous construction of other urban infrastructure, especially the construction of urban public transportation; increase the efforts to facilitate people’s movement; and reduce the traffic barriers for residents in accessing green land resources.

## 6. Conclusions

Using UGS data from 254 cities in China, this paper uses 2SFCA to explore the spatial heterogeneity of accessibility and fairness of UGS under the four search radii of 1 km, 2.5 km, 5 km and 10 km at a community scale. The factor of urban house price is included in the research system and the relationship between urban house price and the fairness of UGS accessibility is explored. The study provides a scientific basis for reasonable adjustments, planning and design of UGS and also explores the use of multi-source data to assist urban planning and management decisions. The study’s main conclusions are as follows.

First, there is a large gap in UGS accessibility among Chinese cities and UGS accessibility remains very poor in some cities at a search radius of 10 km. This highlights the need to conduct in-depth investigation and discussions on the fairness of UGS accessibility between cities.

Second, within a distance that allows for walking and cycling (1 km and 2.5 km search radius, respectively), the degree of inequality with respect to the accessibility of most UGS included in the research system is at the “dangerous” level. There is a significant gap in the distribution of green space resources, with an unbalanced relationship between supply and demand. When the search radius is extended to 5 km and 10 km (driving), the equality of access to UGS is gradually improved, but the accessibility inequality of some cities remains at a “dangerous” level. Generally speaking, from the perspective of residents’ accessibility, the distribution of UGS in China is unfair.

Third, there is a relationship between the fairness of UGS accessibility and housing price. Overall, regional development somewhat increases inequities in UGS accessibility, especially in urban agglomerations with an average housing price of 11,039–23,902 (yuan/m^2^).

Finally, to realize the reasonable allocation of UGS and improve the supply level of urban infrastructure public facilities, our research results highlight three problems related to the accessibility of UGS in China and provides recommendations for UGS development. This study supplements other studies on the large-scale accessibility of UGS and the level of fairness behind it at the community scale. However, this study has some limitations. For example, there are too few samples of green space and community in some cities, which leads to evaluation results that could be more objective. Moreover, human mobility or dynamic population distribution was not well considered in the green space accessibility measurement [72]. In the future, more in-depth research can be conducted when sufficient data are available.

Through in-depth research on this issue, this paper makes the following research contributions. (1) It provides a scientific basis for urban planners to realize the rational layout of urban infrastructure and a sustainable development model. (2) It focuses on the issue of urban environmental justice and provides a new reference related to the topic of green space.

## Figures and Tables

**Table 1:** Criteria of Gini coefficient.

Gini Coefficient Value	<0.3	0.3–0.4	0.4–0.6	>0.6
Level of inequality	Good	Normal	Warning	Dangerous

**Table 2:** Basic data on green space and communities in 254 cities.

	Max	Min	Mean	Std
Number of green spots per city	1220.00	1.00	33.06	8497.34
Area of green spot (hectare)	2693.22	1.00	14.96	2187.80
Number of communities per city	6628.00	1.00	315.06	665,405.40
Housing price (yuan/m^2^)	194,667.000	500.000	16,367.76	315,378,435.09

**Table 3:** *T*-test results of green area and average housing price for cities with ratio > 1 and ratio < 1.

Indicators		1 km	2.5 km	5 km	10 km
Green area	Ratio < 1	280.44	381.44	394.96	407.08
	Ratio > 1	699.49	633.60	692.61	658.48
	*p* value	0.021 **	0.139	0.053 *	0.081 *
Average housing prices	Ratio < 1	10,427.46	15,120.48	13,847.64	13,622.45
	Ratio > 1	20,160.06	18,495.36	24,587.92	26,725.01
	*p* value	0.000 ***	0.000 ***	0.000 ***	0.000 ***

* Significant at the *p* < 0.10 level. ** Significant at the *p* < 0.05 level. *** Significant at the *p* < 0.01 level.

**Table 4:** Relationship between housing price and Gini coefficient of UGS accessibility.

House Price (yuan/m^2^)	1 k	2.5 k	5 k	10 k
500–5500	200.984 ***	293.138 ***	−136.700 ***	−90.623 *
5501–7640	70.428 ***	−2.615	−69.804 *	63.144
7641–11,038	−0.298	117.827	280.930 ***	212.876 **
11,039–23,902	511.583 ***	7048.766 ***	4964.016 ***	1922.889 ***
23,903–194,667	3215.739 *	−58,211.103 ***	−64,405.444 ***	−68,524.843 ***

* Significant at the *p* < 0.10 level. ** Significant at the *p* < 0.05 level. *** Significant at the *p* < 0.01 level.

## Data Availability

Not applicable.

## Appendix A

**Table A1:** City ID, city name and located province and region mentioned in the article.

City ID	City Name	Province	Region
1	Zhangjiakou	Hebei	Eastern region
2	Chengde	Hebei	Eastern region
3	Cangzhou	Hebei	Eastern region
4	Langfang	Hebei	Eastern region
5	Hengshui	Hebei	Eastern region
6	Datong	Shanxi	Central region
7	Yuncheng	Shanxi	Central region
8	Ulanqab	Inner Mongolia	Western region
9	Fushun	Liaoning	Northeast region
10	Dandong	Liaoning	Northeast region
11	Tieling	Liaoning	Northeast region
12	Chaoyang	Liaoning	Northeast region
13	Huludao	Liaoning	Northeast region
14	Siping	Jilin	Northeast region
15	Baicheng	Jilin	Northeast region
16	Jixi	Heilongjiang	Northeast region
17	Jiamusi	Heilongjiang	Northeast region
18	Suihua	Heilongjiang	Northeast region
19	Xuzhou	Jiangsu	Eastern region
20	Nantong	Jiangsu	Eastern region
21	Wenzhou	Zhejiang	Eastern region
22	Jiaxing	Zhejiang	Eastern region
23	Huzhou	Zhejiang	Eastern region
24	Shaoxing	Zhejiang	Eastern region
25	Taizhou	Zhejiang	Eastern region
26	Lishui	Zhejiang	Eastern region
27	Wuhu	Anhui	Central region
28	Bangfu	Anhui	Central region
29	Huangshan	Anhui	Central region
30	Chuzhou	Anhui	Central region
31	Chizhou	Anhui	Central region
32	Quanzhou	Fujian	Eastern region
33	Zhangzhou	Fujian	Eastern region
34	Nanping	Fujian	Eastern region
35	Ningde	Fujian	Eastern region
36	Jiujiang	Jiangxi	Central region
37	Yingtan	Jiangxi	Central region
38	Fuzhou	Jiangxi	Central region
39	Zibo	Shandong	Eastern region
40	Jining	Shandong	Eastern region
41	Taian	Shandong	Eastern region
42	Binzhou	Shandong	Eastern region
43	Heze	Shandong	Eastern region
44	Xinxiang	Henan	Central region
45	Xuchang	Henan	Central region
46	Shangqiu	Henan	Central region
47	Huangshi	Hubei	Central region
48	Shiyan	Hubei	Central region
49	Ezhou	Hubei	Central region
50	Jingzhou	Hubei	Central region
51	Huanggang	Hubei	Central region
53	Zhuzhou	Hunan	Central region
53	Yiyang	Hunan	Central region
54	Foshan	Guangdong	Eastern region
55	Maoming	Guangdong	Eastern region
56	Zhaoqing	Guangdong	Eastern region
57	Qingyuan	Guangdong	Eastern region
58	Yunfu	Guangdong	Eastern region
59	Qinzhou	Guangxi	Western region
60	Yulin	Guangxi	Western region
61	Nanchong	Sichuan	Western region
62	Meishan	Sichuan	Western region
63	Yibin	Sichuan	Western region
64	Yaan	Sichuan	Western region
65	Ziyang	Sichuan	Western region
66	Anshun	Guizhou	Western region
67	Zhaotong	Yunnan	Western region
68	Lincang	Yunnan	Western region
69	Yanan	Shaanxi	Western region
70	Hanzhong	Shaanxi	Western region
71	Jinchang	Gansu	Western region
72	Wuwei	Gansu	Western region
73	Guyuan	Ningxia	Western region
